# Bidirectional communication between the Aryl hydrocarbon Receptor (AhR) and the microbiome tunes host metabolism

**DOI:** 10.1038/npjbiofilms.2016.14

**Published:** 2016-08-24

**Authors:** Agata Korecka, Anthony Dona, Shawon Lahiri, Adrian James Tett, Maha Al-Asmakh, Viorica Braniste, Rossana D’Arienzo, Afrouz Abbaspour, Nicole Reichardt, Yoshiaki Fujii-Kuriyama, Joseph Rafter, Arjan Narbad, Elaine Holmes, Jeremy Nicholson, Velmurugesan Arulampalam, Sven Pettersson

**Affiliations:** 1 Department of Microbiology, Tumour and Cell Biology, Karolinska Institutet, Stockholm, Sweden; 2 Department of Surgery and Cancer, MRC-NIHR National Phenome Centre, Computational and Systems Medicine, Imperial College of London, London, UK; 3 Cardiac Technology Centre, Kolling Institute of Medical Research, Royal North Shore Hospital, University of Sydney, Sydney, NSW, Australia; 4 Institute of Food Research, Norwich Research Park, Norwich, UK; 5 Institute of Molecular and Cellular Biosciences, University of Tokyo, Tokyo, Japan; 6 Department of Biosciences and Nutrition, Novum, Karolinska Institutet, Huddinge, Sweden; 7 Lee Kong Chian School of Medicine, Nanyang Technological University, Singapore, Singapore; 8 SCELSE microbiome centre, Singapore, Singapore

## Abstract

The ligand-induced transcription factor, aryl hydrocarbon receptor (AhR) is known for its capacity to tune adaptive immunity and xenobiotic metabolism—biological properties subject to regulation by the indigenous microbiome. The objective of this study was to probe the postulated microbiome-AhR crosstalk and whether such an axis could influence metabolic homeostasis of the host. Utilising a systems-biology approach combining in-depth ^1^H-NMR-based metabonomics (plasma, liver and skeletal muscle) with microbiome profiling (small intestine, colon and faeces) of AhR knockout (AhR^−/−^) and wild-type (AhR^+/+^) mice, we assessed AhR function in host metabolism. Microbiome metabolites such as short-chain fatty acids were found to regulate AhR and its target genes in liver and intestine. The AhR signalling pathway, in turn, was able to influence microbiome composition in the small intestine as evident from microbiota profiling of the AhR^+/+^ and AhR^−/−^ mice fed with diet enriched with a specific AhR ligand or diet depleted of any known AhR ligands. The AhR^−/−^ mice also displayed increased levels of corticosterol and alanine in serum. In addition, activation of gluconeogenic genes in the AhR^−/−^ mice was indicative of on-going metabolic stress. Reduced levels of ketone bodies and reduced expression of genes involved in fatty acid metabolism in the liver further underscored this observation. Interestingly, exposing AhR^−/−^ mice to a high-fat diet showed resilience to glucose intolerance. Our data suggest the existence of a bidirectional AhR-microbiome axis, which influences host metabolic pathways.

## Introduction

The mammalian body is a mosaic of different microorganisms and eukaryotic cells which share a set of biological and biochemical needs important for growth, body physiology, survival and reproduction (reviewed in reference 1). The gut microbiota, in addition to their ability to process dietary derived material, also influences host responses to xenobiotics,^2^ adding to the growing consensus that factors involved in xenobiotic metabolism could be in intimate partnership with the microbial world. The aryl hydrocarbon receptor (AhR) is a xenobiotic sensor and, belongs to the basic helix–loop–helix Per–Arnt–Sim family and regulates phase I drug-metabolising enzymes from the cytochrome p450 family: Cyp1a1, Cyp1a2 and Cyp1b1.^3^ Apart from well-known man-made pollutants (e.g., 2,3,7,8-tetrachlorodibenzo-*p*-dioxin),^4^ a battery of natural AhR ligands have been discovered. These include kynurenine and planar indoles made during metabolism of tryptophan,^5,6^ such as indole-3-carbinol, which is present in broccoli and cauliflower.^7,8^ AhR is also known to be an important regulator of metabolic and immune processes, both of which are vital for intestinal homeostasis, as well as for optimal coexistence of the host and its microbiome. Ligand-dependent activation of AhR has been shown to abrogate colitis, a disease linked to changes of the gut microbiome homeostasis.^7,9^ More recently, bacterially derived molecules such as phenazines and indole derivates have been shown to work as AhR activators,^9,10^ which implies the existence of a possible microbiome-AhR communication. In this study, host metabolic homeostasis and health has been explored within the context of gut microbiome’s influence on AhR functions.

## Results

### Gut microbiome influence AhR function

**Figure 1:**
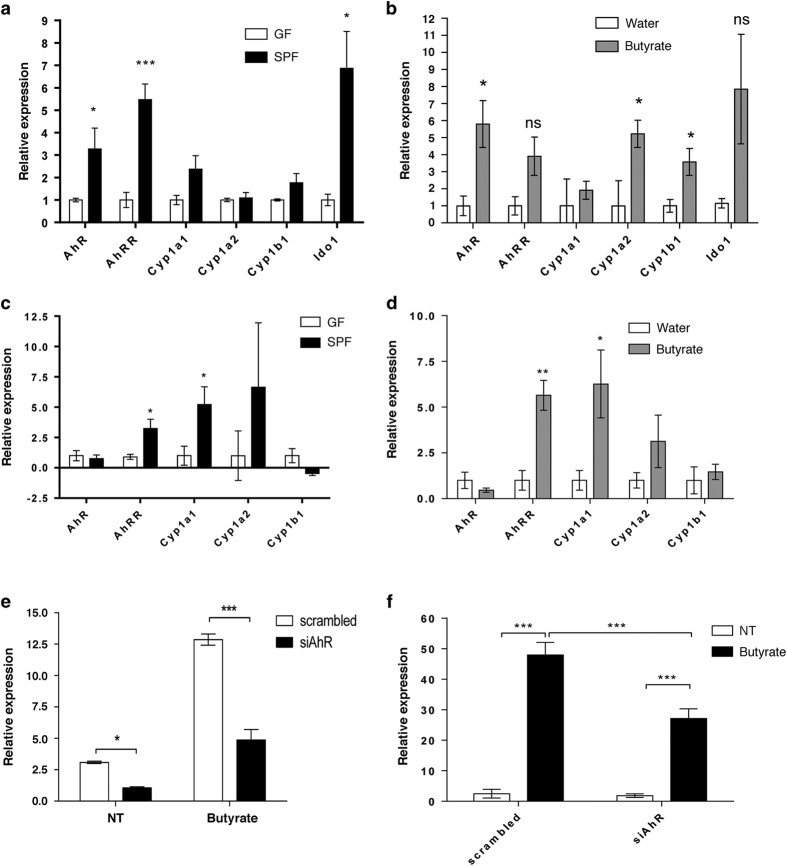
Bacteria and butyrate regulate the expression of AhR and its target genes. (**a**, **b**) Quantitative RT-PCR results depict the expression of *Cyp1a1*, *Cyp1a2*, *Cyp1b1*, *AhRR*, *AhR* and *Ido1* in liver tissue from (**a**) germ-free (GF) and specific pathogen-free (SPF) mice (*n*=5 mice per group), and (**b**) GF mice gavaged with water or butyrate (1 g/kg body weight; *n*=5 mice per group). Quantitative RT-PCR results regarding the expression of *Cyp1a1*, *Cyp1a2*, *Cyp1b1*, *AhRR* and *AhR* in epithelial scrapings from the distal small intestine of (**c**) germ-free (GF) mice and specific pathogen-free (SPF) mice (*n*=5 mice per group) and (**d**) GF mice gavaged with water or butyrate (1 g/kg body weight; *n*=5 mice/group). *P<0.05, **P<0.01 (Student’s *t*-test). (**e**) Quantitative RT-PCR results demonstrate the effects of AhR knockdown by siRNA (siAhR) on *AhR* and (**f**) *Cyp1a1* mRNA expression in HT-29 cells. Cells were transfected with Silencer Select siRNA products directed against AhR (siAhR) or Silencer Select Negative Control #2 siRNA (scrambled). Cells were treated with butyrate (NaB, 2 mmol/l) for 24 h. Control cells (NT) were treated with RPMI medium only. Experiments were performed twice, with biological triplicates, per treatment and per experiment and technical triplicates of each sample for qPCR. Bars and error bars depict the mean±s.e.m. (**a**–**d**) or mean±s.d. (**e**, **f**). Genes of interest were normalised to *Hprt* (**a**), and *18SrRNA* (**b**) and to *β-actin* (**c**–**f**). *P<0.05, ***P<0.001 against GF controls (Student’s *t*-test). *P<0.05, **P<0.01 between indicated bars (two-way analysis of variance (ANOVA)). RT-PCR, reverse transcription PCR.

In the first set of experiments we assessed whether the microbiome or its metabolites could influence AhR function. We compared AhR function in livers of mice carrying a normal bacterial flora (specific pathogen-free, SPF) and that from germ-free (GF) mice. Expression of *AhR* along with the AhR target genes *Cyp1a1* and aryl hydrocarbon receptor repressor (*AhRR*) was higher in the liver of SPF mice than in those of GF mice (Figure 1a). Indoleamine-2,3-dioxygenase (Ido) proteins are key enzymes that control the metabolism of tryptophan to kynurenine, which is a low-affinity ligand for AhR.^6^ The expression of *Ido1* was also induced in the presence of bacterial flora (Figure 1a). The expression of *Cyp1a2*, and *Cyp1b1* though remained unaltered. Short-chain fatty acids (SCFAs), including acetate, propionate and butyrate, are derived through microbiota-driven anaerobic fermentation and are used as an energy source for some cell types, such as colonocytes. Nutrients absorbed from the intestine, including SCFA, are transported to the liver through the enterohepatic circulation and thus can influence metabolic processes in the liver and affect host health. We then assessed how hepatic tissue responded to selected bacterial metabolites. Administration of butyrate to GF mice marginally induced the expression of *AhR and AhRR*. The AhR target genes *Cyp1a2* and *Cyp1b1*, however, responded robustly (Figure 1b).

Furthermore, we confirmed that bacterial signals regulate AhR activity in the intestine as well. We observed significant elevation of *Cyp1a1* and *AhRR* in the intestinal epithelial cells (IECs) of SPF mice than in those of GF mice (Figure 1c). Administration of butyrate to GF mice induced the expression of the *AhRR* and *Cyp1a1*, similarly to the effect observed in the presence of whole bacterial flora (SPF mice; Figure 1d). We also used an *in vitro* system where HT-29 cells were treated with the most prevalent bacterial metabolites, such as acetate, propionate and butyrate (Supplementary Figure 1). Only butyrate was able to induce the expression of both AhR and its target gene Cyp1a1 (Supplementary Figure 1a,b). Propionate could induce AhR expression only, whereas, administration of acetate had no significant effects on the gene expression levels of Cyp1a1 and AhR (Supplementary Figure 1a,b) indicating butyrate to be more efficient to influence AhR activity. To test further whether the effect of butyrate on intestinal epithelial cells is AhR-dependent, we blocked the activity of AhR in HT-29 cells using AhR siRNA (Figure 1e). Butyrate-induced expression of Cyp1a1 was reduced in siRNA treated group, suggesting that butyrate activates the expression of *Cyp1a1* in AhR-dependent manner (Figure 1f). These observations demonstrate that the gut microbiome can activate AhR. Previously, the commensal bacterial strain, *Lactobacillus bulgaricus* OLL1181, has been shown to induce *Cyp1a1* expression in IECs *in vitro* and *in vivo*^11^ further consolidating observations that indigenous bacteria might influence AhR activity. Moreover, microbial metabolites such as the SCFAs (as observed in our study) may affect AhR function indirectly by signal transduction via G-protein-coupled receptors that use SCFA as ligands (GPR41 and GPR43). SCFA may also regulate AhR function through the inhibition of histone deacetylases.^12–14^ Another mode of action maybe through the Toll-like receptor (TLR) signalling, especially via TLR2.^15,16^ In response to oral challenge of AhR ligand benzo(a)pyren, TLR2^−/−^ mice do not show upregulation of the AhR target gene *Cyp1a1* expression.^17^ Furthermore, metabolites produced by the microbiome, owing to their similar aromatic structure, could be considered as endogenous ligands for the AhR, for example phenazines, which are produced by *Enterobacteriacea*, or naphthoquinones, present in a broad range of prokaryotes.^18,19^

### AhR expression influences the gut microbiome composition preferentially in the small intestine

**Figure 2:**
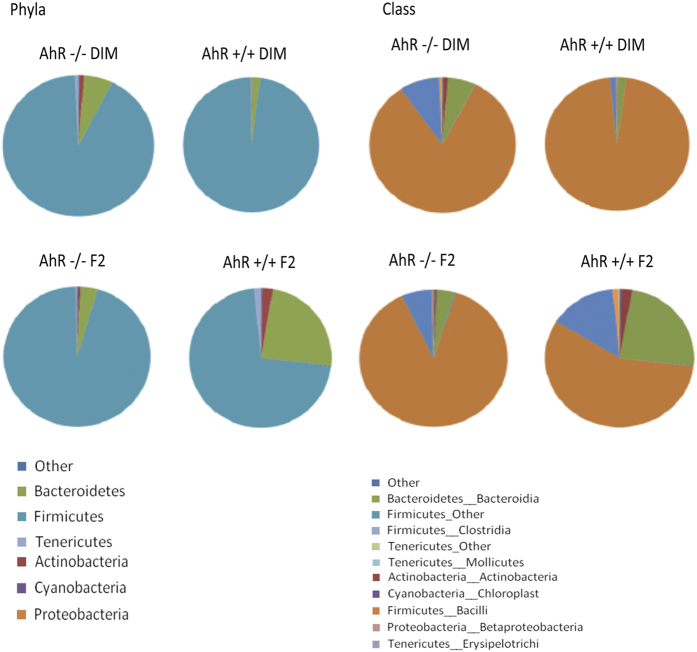
The presence of AhR influences intestinal bacterial composition. The average prevalence of distinct bacterial groups in the small intestine of AhR^+/+^ and AhR^−/−^ mice fed F2 or DIM diets, obtained by 454 sequencing platform. The pie charts depict the composition of the microbiome in the small intestine (*n*=4 AhR^−/−^ DIM-fed mice, *n*=4 AhR^−/−^ F2-fed mice, *n*=5 AhR^+/+^ DIM-fed mice, and *n*=6 AhR^+/+^ F2-fed mice).

Having established a possible microbiome–AhR axis, we next investigated whether AhR expression could influence and shape the intestinal bacterial community. To gain detailed insight into bacterial composition within different compartments of the gastrointestinal tract, we collected colonic and small intestinal contents, as well as faecal samples from AhR knockout (AhR^−/−^) and wild-type (AhR^+/+^) mice for sequencing. AhR is activated by dietary ligands that are present in standard mouse chow (e.g., phenols and tryptophan derivatives). In order to avoid such confounding effects, the offspring of AhR^−/+^ crosses were fed a specially formulated diet depleted of potential AhR ligands (F2 diet) or a F2 diet enriched with a known AhR ligand (DIM diet).^7^ The faecal, colonic, and small intestinal materials were collected from AhR^−/−^ and AhR^+/+^ mice and the composition of bacterial communities were evaluated and compared using 16S ribosomal RNA (rRNA) 454 pyrosequencing. We observed differences in the composition of the microbial communities to the presence or absence of AhR itself, independently of ligand activation (Supplementary Table 1, Figure 2). Bacteroidetes, Actinobacteria and Tenericutes were more prevalent in AhR^+/+^ mice in comparison to the AhR^−/−^ mice on F2 diet. Moreover, small intestines of AhR^+/+^ mice that received a DIM-enriched diet exhibited lower prevalence of Bacteroidetes and higher prevalence of Firmicutes than mice that received F2 chow. Our findings are in accordance with a previous report showing the outgrowth of bacteria belonging to the Bacteroidetes phylum in the small intestines of AhR^−/−^ mice.^8^ This difference in bacterial composition in the small intestine of AhR^+/+^ mice fed F2 versus DIM diet, indicates that the activation of AhR by dietary ligand is able to influence the composition of intestinal bacteria (Supplementary Table 1, Figure 2). Significant differences were also observed within the Firmicutes phylum: bacteria belonging to the class Bacilli were more prevalent in DIM-fed mice, while Clostridia were more prevalent in F2-fed mice. These differences were not pronounced when comparing small intestinal bacterial communities of AhR^−/−^ mice receiving F2 or DIM diets, which emphasises the specificity of response to DIM and excludes the possibility that the food component DIM (which can be treated as a source of bacterial nutrition) directly affects bacterial composition in an AhR-independent manner.

We did not, surprisingly, observe any significant differences in the composition of microbiome in the faeces or colon between the genotypes and diets (Supplementary Tables 2 and 3; Supplementary Figure 2a,b). These results support the notion of regional microbiome-tissue communication that was recently proposed for the crypt region of the intestine.^20^ In addition, this data, raise further concerns regarding the conventional way to profile microbiome status through characterisation of fecal samples. That the faecal bacterial composition might not be fully representative of the communities in various anatomical regions of the gastrointestinal tract has been reported previously.^21^ The distribution and co-localisation of microbiome communities are at present completely unknown and studies to further clarify the prevalence of bacterial phyla, classes, and species within the stomach, small intestine, and colon are highly warranted. Altogether, our results suggest that compromise of AhR function, through genetic modification or lack of ligands, leads to changes in the composition of commensal bacteria within the small intestine.

### AhR regulates energy metabolism

Alterations in gut microbiome influences host metabolism and energy homeostasis. To address the role of AhR in the regulation of energy homeostasis, we measured global changes in metabolic phenotype between AhR^−/−^ and AhR^+/+^ mice by generating metabolic profiles of plasma, liver, and skeletal muscle (Supplementary Figures 3a–c and 4a–c, respectively) using proton nuclear magnetic resonance spectroscopy (^1^H NMR). We compared the levels of identified metabolites in AhR^−/−^ versus AhR^+/+^ animals and observed significant differences in the concentrations of various metabolites (summarised in Table 1) after a 12 h fasting period.

**Figure 3:**
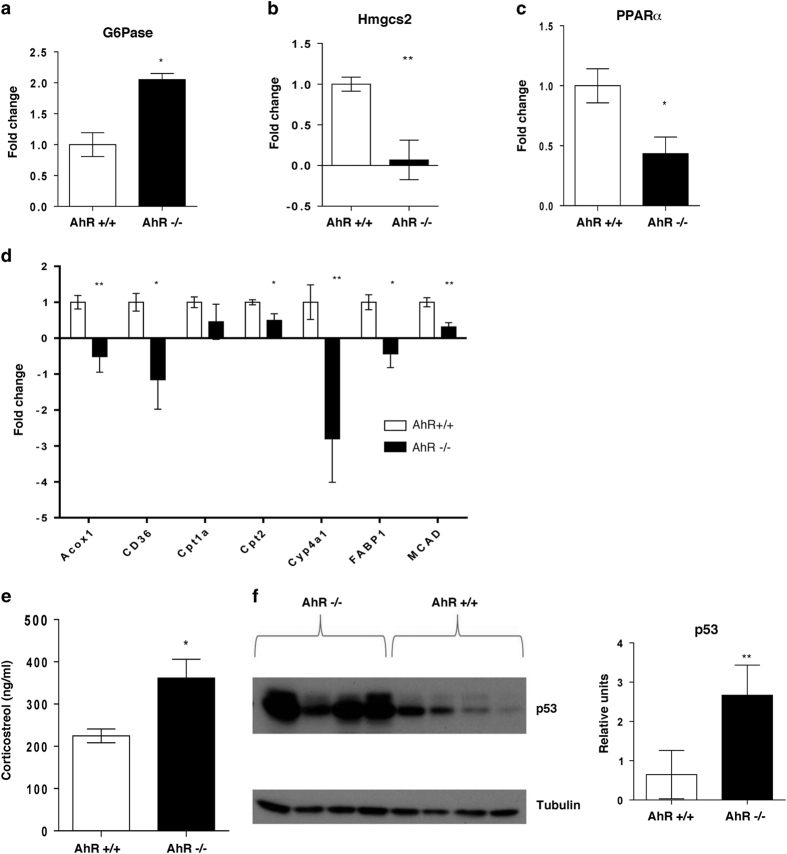
Altered fatty acid and glucose metabolism in AhR^−/−^ mice. Quantitative RT-PCR results regarding the expression of (**a**) glucose-6-phosphatase and (**b**) *Hmgcs2* in liver and *Pparα* (**c**) and genes involved in lipid metabolism (**d**) in the livers of AhR^−/−^ and AhR^+/+^ mice (*n*=4 mice/group). (**e**) Plasma corticosterol levels (measured by ELISA) are higher in AhR^−/−^ mice (*n*=4 mice/group). (**f**) Western blotting (left panel) showing expression of p53 with quantification (right panel) to relative levels of tubulin in the liver of AhR^−/−^ and AhR^+/+^ mice (*n*=4 mice/group). Bars and error bars depict the mean s.e.m. Gene expression for **a**, **b** was normalised to *18SrRNA*, whereas for **c**, **d** normalised to *Hprt*. *P<0.05; **P<0.01 against AhR^+/+^ mice (Student’s *t*-test).

Glucose levels in both plasma and liver were found to be lower in the AhR^−/−^ mice than in AhR^+/+^ mice, while levels of lactate (the main product of glycolysis) were elevated in the plasma and the skeletal muscle of AhR^−/−^ mice. Lactate, together with alanine acts as important substrate for gluconeogenesis during fasting. Notably, levels of alanine were lower in AhR^−/−^ muscle and liver. Increased release of lactate and alanine into blood, as observed in AhR^−/−^ probably indicate that glucose-utilising peripheral tissues catabolise glucose and hence allow it to be utilised as gluconeogenic precursors in the liver undermining the metabolic switch to gluconeogenesis to provide energy to the system during fasting. Glycerol is another known substrate for gluconeogenesis. Glycerol levels were found to be higher both in the plasma and liver of AhR^−/−^ mice, indicating that these metabolites might also be used as substrates for gluconeogenesis. On the basis of these observations, we queried whether gluconeogenesis was altered in AhR^−/−^ mice liver by checking the gene expression level of glucose-6-phosphatase (G6Pase), the final enzyme in the gluconeogenesis pathway in the liver. Indeed, the level of G6Pase was higher in AhR-deficient mice (Figure 3a) confirming that gluconeogenesis is induced in AhR^−/−^ mice probably to maintain blood glucose levels to sustain energy metabolism of other glucose-dependent tissues in the fasted state.

Another striking difference was the lower level of ketone bodies (3-hydroxybutyrate) in the plasma and skeletal muscle of AhR^−/−^ mice (Table 1). During fasting, ketone bodies are produced as a product of fatty acid oxidation or metabolism of certain amino acids. The liver synthesises and releases ketone bodies, primarily 3-hydroxybutyrate, to be used as fuel by peripheral tissues. Decreased levels of ketone bodies in both plasma and muscle reflect that AhR^−/−^ mice are somehow impaired of utilising fatty acid oxidation as their source of fuel. This was further evaluated by observing lower levels of expression of *Hmgcs2,* the main enzyme controlling ketone body production (Figure 3b), and of various genes involved in fatty acid transport and metabolism. Reduced hepatic *Pparα* expression level was also observed in AhR^−/−^ mice compared to AhR^+/+^ mice (Figure 3c). We subsequently also observed that the mRNA expression levels of other genes involved in fatty acid transport and metabolism (*Cd36*, *Fabp1*, *Acox1*, *Cpt1a*, *Cpt2*, *Cyp4a1* and *Mcad*), were generally downregulated in AhR^−/−^ mice (Figure 3d). Impaired fatty acid oxidation and enhanced gluconeogenesis indicates that AhR^−/−^ mice might be experiencing metabolic stress, which is reflected by increased levels of corticosterol in the blood. Indeed, as expected higher levels of glucocorticoids in the plasma of AhR^−/−^ mice were observed (Figure 3e). Elevated levels of glucocorticoids, as well as cellular stress, is known to accumulate and stabilise p53, a master regulator known to promote cellular survival under energy shortage conditions.^22–24^ Most interestingly, we did observe elevated levels of p53 protein in the livers of AhR^−/−^ mice (Figure 3f) further underscoring the metabolic duress in AhR^−/−^ mice.

**Figure 4:**
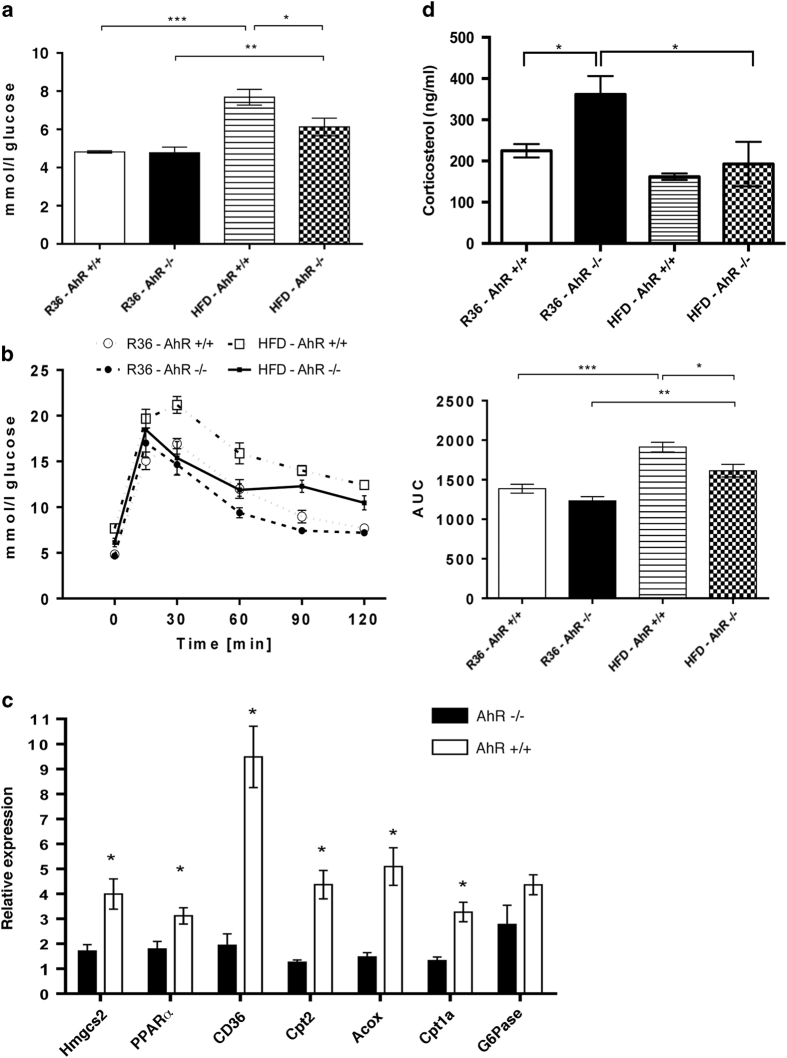
A high-fat diet reduces nutritional stress on AhR^−/−^ mice. Fasting glucose levels (**a**) and the oral glucose tolerance test (R36 AhR^+/+^, *n*=5 and AhR^−/−^, *n*=4; HFD AhR^+/+^, *n*=6 and HFD AhR^−/−^, *n*=4) (**b**), left panel: glucose changes over time; right panel: area under the curve (R36 AhR^+/+^, *n*=5 and AhR^−/−^, *n*=4; HFD AhR^+/+^, *n*=6 and HFD AhR^−/−^, *n*=4). (**c**) Quantitative RT-PCR results regarding the expression of *Hmgcs2*, *PPARα*, *CD36*, *Cpt2*, *Acox*, *Cpt1a*, and *G6Pase* in the livers of AhR^−/−^ and AhR^+/+^ mice in response to high-fat diet (HFD) (R36 AhR^+/+^, *n*=5 and AhR^−/−^, *n*=4; HFD AhR^+/+^, *n*=6 and HFD AhR^−/−^, *n*=4). Gene expression was normalised to *Hprt*. *P<0.05, **P<0.01 against AhR^+/+^ mice (Student’s *t*-test). (**d**) Effect of chow (R36) and HFD on plasma corticosterol levels in AhR^−/−^ and AhR^+/+^ mice (R36 AhR^+/+^, *n*=5 and AhR^−/−^, *n*=4; HFD AhR^+/+^, *n*=6 and HFD AhR^−/−^, *n*=4). Corticosterol level was measured in plasma by ELISA. Bars and error bars depict the mean±s.e.m.

In order to understand the metabolic limitations of the AhR-deficient mice, we challenged these mice with a diet rich in fat. For eleven consecutive weeks, AhR^−/−^ and AhR^+/+^ mice were fed a semi-synthetic chow in which 40% of calories are derived from fat (high-fat diet, HFD). No significant differences in weight gain (Supplementary Figure 5a), chow intake (Supplementary Figure 5b), or fasting insulin levels (Supplementary Figure 5e) were observed between the two genotypes. Interestingly, at the basal condition we observed that the body weight of AhR^−/−^ mice is significantly lower than the AhR^+/+^ mice (Supplementary Figure 5c). However, this difference in body weight no longer exists once the mice were fed HFD for eleven weeks (Supplementary Figure 5d). A significant increase in body weight was observed in the AhR^+/+^ and AhR^−/−^ mice after 11 weeks of HFD treatment in comparison with the respective chow-treated groups (Supplementary Figure 5d). However, there was no significant difference in the food intake between the AhR^+/+^ and AhR^−/−^ mice on chow diet or on HFD (Supplementary Figure 5b). Surprisingly, we observed that AhR^−/−^ mice exhibited lower fasting glucose levels (Figure 4a; Table 2) and improved glucose tolerance (Figure 4b), compared with AhR^+/+^ mice indicating partial protection against diet-induced glucose intolerance in AhR^−/−^ mice. Furthermore, the expression of glucose-6-phosphatase seemed to be lower though not statistically significant in AhR^−/−^ mice livers compared with AhR^+/+^ mice when fed HFD (Figure 4c). This is in striking contrast to our observations in fasted conditions under normal chow feeding. Thus higher hepatic glucose levels (Table 2) along with higher gluconeogenic precursors such as alanine and lactate in HFD fed AhR^−/−^ mice liver reflects a possible mode to control and maintain peripheral glucose levels in response to HF feeding. This might possibly be due to better glucose disposal to peripheral tissues. From these observations it is evident that AhR is instrumental in the dynamic regulation of whole-body glucose homeostasis depending on nutrient availability and energy demand of the host. However, metabolic profiling of plasma in HFD conditions revealed similar differences for 3-hydroxybutyrate between AhR^−/−^ and AhR^+/+^ mice as were observed for normal chow-fed mice (summarised in Table 2). Consequently, the expression of *Hmgcs2* and *Pparα* remained lower along with other genes involved in fatty acid transport and oxidation in livers of AhR^−/−^ mice (Figure 4c) signifying impaired hepatic lipid metabolism in these mice. HF feeding also abrogated the differences in the levels of plasma corticosterol in AhR^−/−^ mice to the level observed in AhR^+/+^ mice (Figure 4d) reflecting that their modulations are indeed in response to the metabolic milieu that could be altered as and when energy demand of the system changes. We also observed high expression levels of p53 in AhR^−/−^ mice in comparison to AhR^+/+^ mice when challenged with HFD (Supplementary Figure 5f), though not statistically significant. The induced p53 is probably a reflection of the metabolic stress these mice encounter, as also observed in AhR^−/−^ mice in basal condition on chow diet (Figure 3f). Hence, it seems that the AhR^−/−^ mice is likely at a metabolic advantage through enhanced gluconeogenesis in liver during fasting to regulate hypoglycemia. However, with dietary challenge the AhR^−/−^ mice is probably more efficient in disposal of glucose load to peripheral tissues as well as restricting gluconeogenesis, preventing HFD induced glucose intolerance.

## Discussion

In the present study, we have identified a bidirectional microbiome-AhR axis that influences host metabolism in the liver and ketone body production. Our findings showing that production of ketone bodies can be regulated by AhR implies an AhR-dependent feed-forward mechanism to secure nutrients to the host under conditions of starvation. Our observations also suggest a novel but less understood role of AhR in the modulation of gut microbiome composition in the small intestine. Such changes in the microbiome possibly impart metabolic consequences and may contribute to deregulated energy metabolism. However, an altered immune system imparting the changes seen in the microbiota composition of AhR^−/−^ mice cannot be ruled out. Indeed, the reported elevation of inflammation circuits in AhR^−/−^ mice supports such a mechanism. In a recent study the ketone metabolite beta-hydroxybutyrate, was shown to suppress NLRP3-driven inflammation under nutritional constrain conditions.^25^ Thus, AhR-mediated regulation of ketone body production illustrates the intricate interplay between inflammation and metabolism where this metabolic product may act as an immunomodulatory currency.

Most well-known ligands for the AhR, including biphenyls, phenylalanine hydroxylases, aromatic amines and dioxins, are lipid-soluble molecules.^4^ These substances, also known as persistent organic pollutants, accumulate in the white adipose tissue and are released from this tissue together with lipids.^26^ Persistent organic pollutants have endocrine disruptive properties and can interfere with the activity of many nuclear receptors, resulting in profound alterations of hormonal balance.^27^ We speculate that lack of AhR, one of the main proteins that orchestrate the breakdown of these dangerous substances, initiates transcriptional and translational changes in the liver in order to protect it from the toxic effects of persistent organic pollutants. Possible protective responses include the downregulation of lipid transport to the liver by decreasing the expression of *Cd36* and *Fabp1* and also invoking cellular responses to stress through the regulation of gluconeogenesis, by increasing hepatic glucose production and the expression of glucose-6-phosphatase in the liver.^28^

Disturbances in glucose and fatty acid metabolism may lead to serious metabolic problems, including type II diabetes and obesity-related co-morbidities. Quantitative trait locus analysis of dietary obesity in C57BL/6 and129P3/J F2 mice revealed that the *AhR* gene is one of seven candidate genes associated with increased body weight.^29^ Moreover, a shift in the ratio between Bacteroidetes and Firmicutes in the intestine has been linked with development of obesity in both mice and humans.^30–34^ We did not observe a difference in weight gain between AhR^−/−^ and AhR^+/+^ mice on HFD. However, we did observe that the AhR^−/−^ mice were partially protected against diet-induced glucose intolerance. Whether this protection is due to their altered microbiome composition in the small intestine in the AhR^−/−^ mice remains to be seen. In order to demonstrate cause or consequence effects, extensive additional experiments will have to be done, but is beyond the scope of this study.

Our data suggest that bilateral communication links the microbiome to AhR, an evolutionarily conserved environmental sensor in many eukaryotes, impacting immune and energy homeostasis. Dynamic changes in the gut microbiome may confer metabolic and developmental consequences to the host through AhR. The current study has established such associations between microbiome-AhR crosstalk. Further experiments are certainly required to reveal the more precise mechanisms and to identify the set of selected microbial metabolites that may account for the observed metabolic effects. Finally, AhR resides within a family of drugable receptors with an abundance of putative ligands, making it an attractive target for future treatment of metabolic and other disorders.

## Materials and methods

### Animals

All mice (C57Bl6/J background) were maintained on autoclaved R36 Lactamin chow (Lactamin, Stockholm, Sweden) on a 12-h light/dark cycle. In the HFD experiment, mice received a semi-synthetic fat-rich chow R638 (40% of calories from fat) from Lantmännen, Sweden. For experiments with bacterial flora sequencing, mice received semi-synthetic F2 chow depleted of any naturally occurring AhR ligands or F2 diet enriched in DIM, a known AhR ligand, from postnatal day 1. Mice were assigned to receive F2 or DIM diet at random. F2 and DIM was a generous gift from Fujii—Kurijama (University of Tokyo, Tokyo, Japan). This food was introduced at day P1 (to avoid any developmental problems caused by lack of AhR ligands *in utero*) and mice were randomly assigned to receive F2 or DIM diets. The AhR^−/−^, AhR^+/+^ and AhR^−/+^ offspring were co-housed and genotyped at the 5 weeks of age. The faecal, colonic and small intestinal material was collected from AhR^−/−^ and AhR^+/+^ mice when they reached the 8 weeks of age. Only healthy male mice of similar age and weight were used in these experiments after which wild type and knockout mice were randomly assigned to different treatments. For experiments involving butyrate treatment, GF mice (8–10 weeks of age) were gavaged with water or butyrate (1 g/kg body weight) and killed after 72 h of treatment. Experimental protocol was similar to previously published treatment dose and schedule.^35^

Mice were killed by cervical dislocation at the end of experiment. All protocols involving the use of animals were approved by the Regional Animal Research Ethical Board, Stockholm, Sweden (Stockholms norra djurförsöksetiska nämnd), following proceedings described in EU legislation (Council Directive 86/609/EEC). Animal husbandry was in accordance with Karolinska Institutet guidelines and approved by the above-mentioned ethical board (Ref: N 100/10 and 299/12). Animal experiments adhered to 3R policy to ensure minimum numbers of animals were used to maximise data mining.

AhR^+/−^ mice were initially obtained from CLEA, Japan, and subsequently crossbred to obtain AhR^+/+^, AhR^+/−^ and AhR^−/−^ genotypes. AHR^+/+^ and AhR^−/−^ mice were crossed to C57Bl/6J background. The generation of AhR^+/−^ has been described previously.^36^ Mice were maintained under SPF conditions. Experiments with GermFree mice (GF) were performed at the Core Facility for Germfree Research (CFGR) Karolinska Institutet, Stockholm, Sweden.

### Glucose tolerance testing and insulin measurements

Glucose tolerance tests were performed with the use of Roche Acuvue glucometer and adequate strips. For each test, a blood drop was collected from the tip of the tail. Blood was collected after an overnight (12 h) fasting period, and then 15, 30, 60, 90 and 120 min after oral glucose administration (2 g/kg body weight). Insulin levels in mice sacrificed after overnight fasting (12 h), were measured in serum by ELISA (Millipore, Billerica, MA, USA) post mortem.

### Cell lines, culture conditions and treatments

The human epithelial cell line HT-29 (HBT-11) (ATCC, Rockville, MD, USA) was cultured in RPMI 1640 media supplemented with 10% heat inactivated foetal bovine serum (both from Invitrogen, Carlsbard, CA, USA). Cells were maintained in a 5% CO_2_ humidified atmosphere at 37 °C. Cell morphogenesis was monitored microscopically. To prevent contact inhibition, cell densities for each experiment did not exceed 80%. Dimethyl sulfoxide and sodium butyrate were purchased from Sigma Aldrich (St Louis, MO, USA), 3,4-dimethoxyflavone was from Cayman Chemicals (Ann Arbor, MI, USA). Inhibition experiments using AhR inhibitor 3,4-dimethoxyflavone (10 μmol/l) were performed by pretreating cells with the inhibitor or vehicle (dimethyl sulfoxide) for 1 h before stimulating cells with sodium butyrate. Downregulation of AhR transcripts in HT-29 cells were achieved by SMART Pool siRNA products directed against AhR (ThermoScientific). Controls were transfected with Silencer SMART Pool Non-Targeting siRNA (ThermoScientific, Waltham, MA, USA). Transfection was carried out according to the manufacturer’s protocol using DharmaFECT 4 (ThermoScientific) reagent (final concentration, 0.3%), with a final siRNA concentration of 40 nM. Cells were treated with acetate (10 mmol/l), propionate (5 mmol/l) or butyrate (2 mmol/l) for 24 h as previously published.^37^ Control cells (NT) were treated with RPMI medium only. All *in vitro* experiments were performed twice, with three biological replicates per treatment and per experiment.

### RNA extraction and RT-qPCR

RNA was isolated using the Qiagen RNeasy Mini kit according to the manufacturer's instructions. cDNA was synthesised with SuperScript II (Invitrogen). OligodT primers were used in the presence of RNaseOUT reagent (Invitrogen). One microgram of RNA was used per reaction. Complementary DNA (cDNA) was diluted 1:5 and then 1 μl was used for each quantitative PCR (qPCR) reaction. qPCR was performed using SYBRGreen reagent (Applied Biosystems, Carlsbard, CA, USA ) and gene-specific primers (Supplementary Table 4). Reactions were performed with the use of an Abi Prism 7500 (Applied Biosystems) thermal cycler. Housekeeping genes were carefully selected for each experiment so that their expression levels did not exhibit significant differences between treatments. Relative expression was calculated using the formula 2^−ΔΔCt^. The average from the controls was taken as 1, and fold change for each treatment was calculated accordingly. Each sample was tested in triplicate for qPCR.

### Western blotting

Livers were collected from fasting mice (12 h) and perfused with PBS prior to dissection. Tissue was lysed in RIPA buffer (150 mmol/l NaCl, 50 mmol/l Tris pH=8, 0.25 mmol/l EDTA, 1% sodium deoxycholate, 1% Triton X-100, 5 mmol/l sodium fluoride; 400 mmol/l sodium vanadate; 1 mmol/l phenylmethanesulphonylfluoride, 1 mmol/l dithiothreitol; 1× Complete Protease Inhibitors) (Roche, Mannheim, Germany). The anti-p53 antibody was from Cell Signalling (2524), anti-mouse secondary peroxidase conjugated antibody from DAKO A/S (P0447), and β-tubulin primary antibody conjugated with horse-radish peroxidase from Abcam (ab21058). Detection was conducted by chemiluminescence (BioRad, Hercules, CA, USA). Expression of p53 was quantified against β-tubulin using Image-J software (NIH, Bethesda, MD, USA).

### Statistical analysis

Statistical analyses were performed using GraphPad Prism 6 statistical software (La Jolla, CA, USA). For analysis with multiple groups, One-way or Two-way analysis of variance tests were performed where relevant. Unpaired *t*-test (two-tailed) was performed when observations between two groups were compared. *P*<0.05 was considered statistically significant unless otherwise stated. Values were expressed as mean±s.e.m.

### NMR metabolic profiling

#### Plasma sample preparation

Sample preparation and acquisition methods were annotated from previously published methods.^38,39^ Aliquots of mouse plasma (100 μl) were mixed with 500 μl of saline solution (0.9% NaCl in D_2_O), incubated for 10 min at room temperature, and then centrifuged at 13000 rpm for 10 min in order to remove insoluble material. Supernatants were transferred into 5 mm NMR tubes for ^1^H NMR analysis.

#### Preparation of aqueous tissue extracts

For liver and muscle analysis, an amount of each sample was weighed out (~200 mg liver; ~50 mg muscle) and added to 1.5 ml of 50:50 water/methanol. Samples were incubated on dry ice for a few minutes before adding 30–40 high-density 1-mm zirconia beads. The samples were then homogenised in a bead beater (Precellys 24) for 3 cycles (5 min each); samples were kept on dry ice between cycles. Next, the samples were centrifuged at 13,000 r.p.m. for 10 min, and 500 μl aliquots were transferred into a separate Eppendorf tube. The pellet was dried and retained for the later organic extraction. Protein was precipitated from the aqueous phase by adding 1 ml methanol, vortexing for 3 min (Multimixer, Thomas Scientific, Swedesboro, NJ, USA, and incubating the samples at −20 °C overnight. Aliquots of 500 μl were then taken from the supernatant, dried in a speed vacuum overnight at room temperature, and subsequently frozen at −80 °C. Before NMR acquisition, samples were resuspended in 550 μl phosphate buffer solution (0.2 M Na_2_HPO_4_/0.04 mol/l NaH_2_PO_4_, pH=7.4 with 0.1% sodium azide and 1 mmol/l 3-trimethylsilyl-1-[2,2,3,3,-^2^H_4_] propionate in D_2_O) and transferred to a 5 mm NMR tube for analysis.

#### Acquisition of ^1^H NMR spectra

^1^H NMR spectra were acquired with a Bruker Avance 600 MHz spectrometer (Bruker Biospin, Karlsruhe, Germany) operating at 600.13 MHz for ^1^H at 300 K. It was equipped with a 5 mm broadband inverse configuration probe. Both plasma and tissue extracts were analysed with a water-suppressed 1D NMR spectrum using the NOESYPRESAT pulse sequence (256 transients). Irradiation of the solvent (water) resonance was applied during presaturation delay (2.0 s) for all spectra and for the water-suppressed 1D NMR spectra also during the mixing time (0.1 s). The pulse sequence parameters, including the 90° pulse (~12 μs), pulse frequency offset (~2,800 Hz), receiver gain (~200), and pulse powers, were optimised for each sample set run. The spectral width was 20 p.p.m. for all spectra. The NMR was processed with 1.0 Hz exponential line broadening prior to Fourier transformation. Fourier transformations were collected with ~32,000 real data points.

#### NMR spectral data pre-processing

Data (−1.0 to 10.0 p.p.m.) were imported into MATLAB 7.0 software (MathWorks, Natick, MA, USA), in which they were automatically phased, baseline corrected and referenced to the 3-trimethylsilyl-1-[2,2,3,3,-^2^H_4_] propionate peak (0.00 p.p.m.) using scripts written in-house. To reduce analytical variation between samples, the residual water signal (4.67–4.98 p.p.m.) was truncated from the data set. To enable separate normalisation to total area and use of probabilistic quotient (median fold-change) methods, each spectrum was set to have a unit total intensity such that each data point was expressed as a fraction of the total spectral integral.^40^ Endogenous plasma metabolites and metabolites extracted from liver and muscle tissues were assigned by referring to data from published literature,^41–44^ as well as to in-house and online databases.

#### Statistical methods and software

After the pre-processing of the NMR data, multivariate statistical analysis was performed using both Matlab R2013b (MathWorks, Natick, MA, USA) and SIMCA-P 13.0 software packages (Umetrics, Umeå, Sweden). Principal components analysis (PCA) was performed using univariate scaling. The first two components of variation were plotted against one another to access the inter-cohort variation across the global metabolic profile. Orthogonal partial least squares discriminant analysis (OPLS-DA) using both univariate and mean centred scaling was employed to identify specific metabolites pertaining to a particular sample group.^45^ All OPLS models were validated using random permutation testing of the supervised model.

### High-throughput sequencing of bacterial content

#### DNA extraction and sequencing of 16S rRNA gene regions

DNA was extracted from each sample using the FastDNA Spin Kit for soil (MP Biomedicals, Leicester, UK). A modified protocol was employed as described.^46^ For each sample, the V4 and V5 regions of the 16S rRNA genes were amplified using the universal primers U515F (5′- GTGYCAGCMGCCGCGGTA-3′) and U927R (5′- CCCGYCAATTCMTTTRAGT-3′). The forward fusion primer also contained the GS FLX Titanium primer A, library Key (5′- CCATCTCATCCCTGCGTGTCTCCGACTCAG-3′), and 10-bp multiplex identifiers (MID) (Roche Diagnostics, West Sussex, UK). The reverse fusion primer included the GS FLX Titanium primer B and library key (5’- CCTATCCCCTGTGTGCCTTGGCAGTCTCAG-3′) identifiers. Amplification conditions, sample pooling, preparation and sequencing on the GS FLX titanium platform were undertaken as previously described.^47^

#### Data analysis

Raw 16S rDNA sequences were processed in QIIME^48^ version 1.5.0 using default parameters. Sequences were removed from the analysis if they were <350 or >450 base pairs, were of low quality, contained ambiguous bases or if there were mismatches in the barcode or forward sequencing primer. The reverse sequence primer was removed. Remaining sequences were clustered into operational taxonomic units using UCLUST^49^ at 97% sequence identity. A representative sequence for each operational taxonomic unit was chosen and assigned taxonomy using the RDP classifier^50^ and Greengenes (February 2011 release).^51^ Sequences were rarefied to 3568 to remove bias caused by heterogeneity in the number of sequences for each sample. The Mann–Whitney *U*-test was used for statistical analysis of the samples.

## Figures and Tables

**Table 1:** List of metabolites that were found to be different in AhR^+/+^ and AhR^−/−^ mice (*n*=4 mice/group)

*Lower in AhR^−/−^*		*Higher in AhR^−/−^*	
*Metabolite*	*Correlation coefficient*	*Metabolite*	*Correlation coefficient*
*Plasma*			
Methylmalonate	0.8	Tyrosine	0.9
Glucose	0.5	Creatine	0.9
3-Hydroxybutyrate	0.7	Alanine	0.8
		Lactate	0.8
		Citrate	0.8
		TMAO	0.7
		Glycerol	0.7
			
*Liver*			
Inosine	0.8	Creatine	0.9
Choline	0.8	TMAO	0.7
Leucine	0.6	Betaine	0.6
Valine	0.6	Glycerol	0.5
Isoleucine	0.6	Taurine	0.3
Glutamate	0.5	Acetate	0.3
Glucose	0.3		
Alanine	0.3		
Lactate	0.3		
			
*Skeletal muscle*			
Taurine	0.6	Lactate	0.6
3-hydroxybutyrate	0.7		
Alanine	0.8		
Isoleucine	0.8		

Abbreviation: AhR, aryl hydrocarbon receptor; TMAO, trimethylamine N-oxide.

**Table 2:** List of metabolites which levels were found to be different in AhR^+/+^ and AhR^−/−^ mice fed high-fat diet

*Lower in AhR^−/−^*		*Higher in AhR^−/−^*	
*Metabolite*	*Correlation coefficient*	*Metabolite*	*Correlation coefficient*
*Plasma*			
Methylmalonate	0.8	Tyrosine	0.9
Glucose	0.5	Creatine	0.9
3-hydroxybutyrate	0.7	Alanine	0.8
		Lactate	0.8
		Citrate	0.8
		TMAO	0.7
		Glycerol	0.7
			
*Liver*			
Choline	0.7	Creatine	0.8
Formate	0.7	Glucose	0.5
3-hydroxybutyrate	0.5	Glycerol	0.5
Inosine	0.3	Alanine	0.4
		Lactate	0.3
			
*Skeletal muscle*			
		Creatine	0.9
		Taurine	0.8
		Lactate	0.6
		Anserine	0.9
		Carnosine	0.8

Abbreviation: AhR, aryl hydrocarbon receptor; TMAO, trimethylamine N-oxide .
