# Supplementary material for: Bidirectional communication between the Aryl hydrocarbon Receptor (AhR) and the microbiome tunes host metabolism
Source: NPJ Biofilms Microbiomes. 2016 Aug 24;2:16014–. doi: 10.1038/npjbiofilms.2016.14 (PMC5515264; doi:10.1038/npjbiofilms.2016.14)
Supplement: Supplementary Information [file npjbiofilms201614-s1.doc]

**Supplementary information:**

**Supplementary Figure Legends:**

**Supplementary Figure 1. Bacterial metabolites affect AhR expression. (a).** Quantitative RT-PCR results for AhR and **(b)** Cyp1a1 expression in HT-29 cells treated for 24 hours with acetate (10mM), propionate (5mM), and butyrate (2mM). n=4 per group, bars and error bars show means +/- SEM. ****, p<0,0001 against the control group (One-Way ANOVA followed by Dunnett’s multiple comparison test) Experiments were performed twice, with biological triplicates, per treatment and per experiment and technical triplicates of each sample for qPCR.

**Supplementary Figure 2. Comparison of bacterial composition from faecal and colonic material.** Average prevalence of distinct bacterial groups in the faeces **(a)** and colonic content **(b)** of AhR^+/+^ and AhR^-/-^ mice fed F2 or DIM diets. Data obtained by 454 sequencing platform. The wheel charts depict the composition of the microbiome in the small intestine with respective coloured legends. n=4 AhR^-/-^ DIM-fed mice, n=4 AhR^-/-^ F2-fed mice, n=5 AhR^+/+^ DIM-fed mice, and n=6 AhR^+/+^ F2-fed mice.

**Supplementary Figure 3. Comparisons of ^1^H NMR metabonomics-derived metabotypes of AhR^+/+^ and AhR^-/-^ mice.** PCA analysis of plasma (**a**), aqueous liver extracts (**b**), and aqueous muscle extracts (**c**) for AhR^+/+^ and AhR^-/-^ mice (n=4 mice/group).

**Supplementary Figure 4. Metabolic spectra and assignment of (a) plasma, (b) aqueous liver extract, and (c) aqueous muscle extract from AhR^-/-^ and AhR^+/+^ mice** (n=4 mice/group)**.**

**Supplementary Figure 5. Basic metabolic parameters of AhR^-/-^ and AhR^+/+^ mice on the high-fat diet.** **(a)** Body weight changes over time. **(b)** Food intake after 11 weeks of HFD intervention (R36 AhR^+/+^ n=5 and AhR^-/-^ n=4; HFD AhR^+/+^ n=6 and AhR^-/-^ n=4). **(c)** Body weight at basal condition before HFD intervention and **(d)** after 11 weeks of HFD intervention (R36 AhR^+/+^ n=5 and AhR^-/-^ n=4; HFD AhR^+/+^ n=6 and AhR^-/-^ n=4). **(e)** Insulin levels after 11 weeks of HFD intervention (R36 AhR^+/+^ n=5 and AhR^-/-^ n=4; HFD AhR^+/+^ n=6 and AhR^-/-^ n=4). **(f)** Western blotting (*left panel*) showing expression of p53 with quantification to relative levels of Tubulin (*right panel*) in the liver of AhR^-/-^ and AhR ^+/+^ mice on 11 weeks of HFD (HFD AhR^+/+^ n=5 and AhR^-/-^ n=4; data analysed by Student’s t-test; unpaired, two-tailed). Bars and error bars depict the mean +/- SEM. *p<0.05, **p<0.01 and *** p<0,001 between indicated bars (data analysed with One way ANOVA followed by Tukey’s multicomparison test).

**Supplementary Figures:**

**
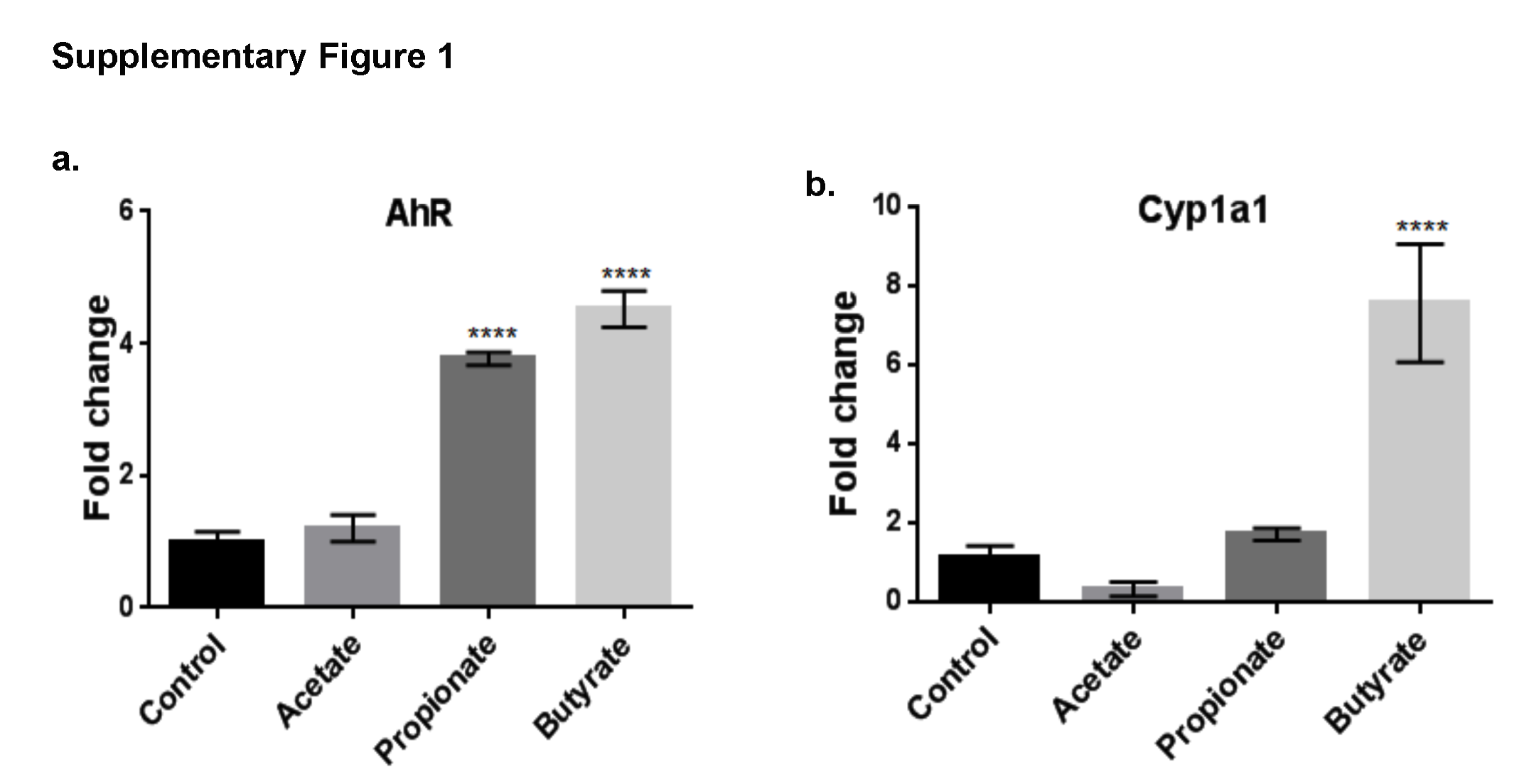
**

**
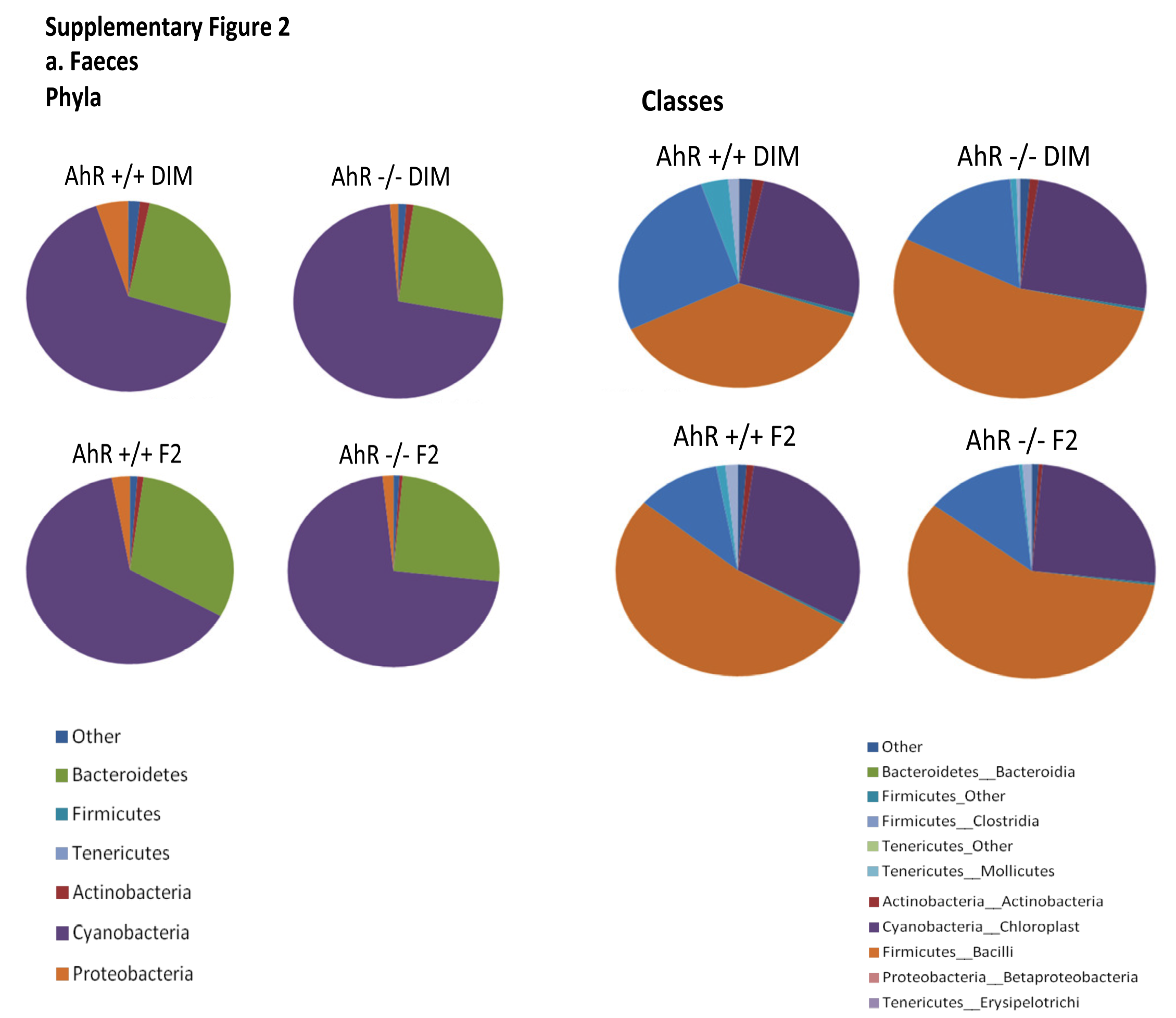
**

**
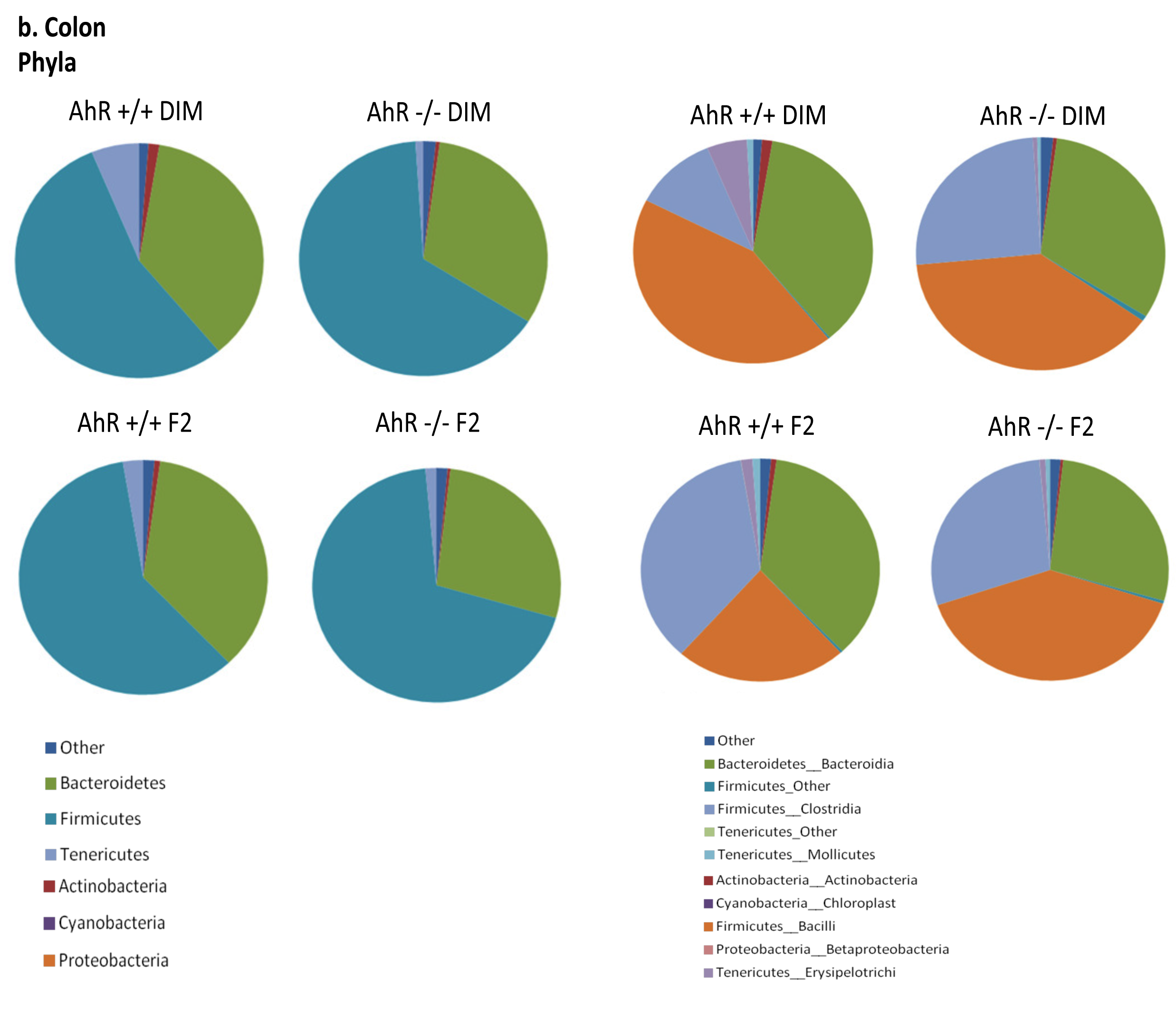
**

**Supplementary Figure 3**


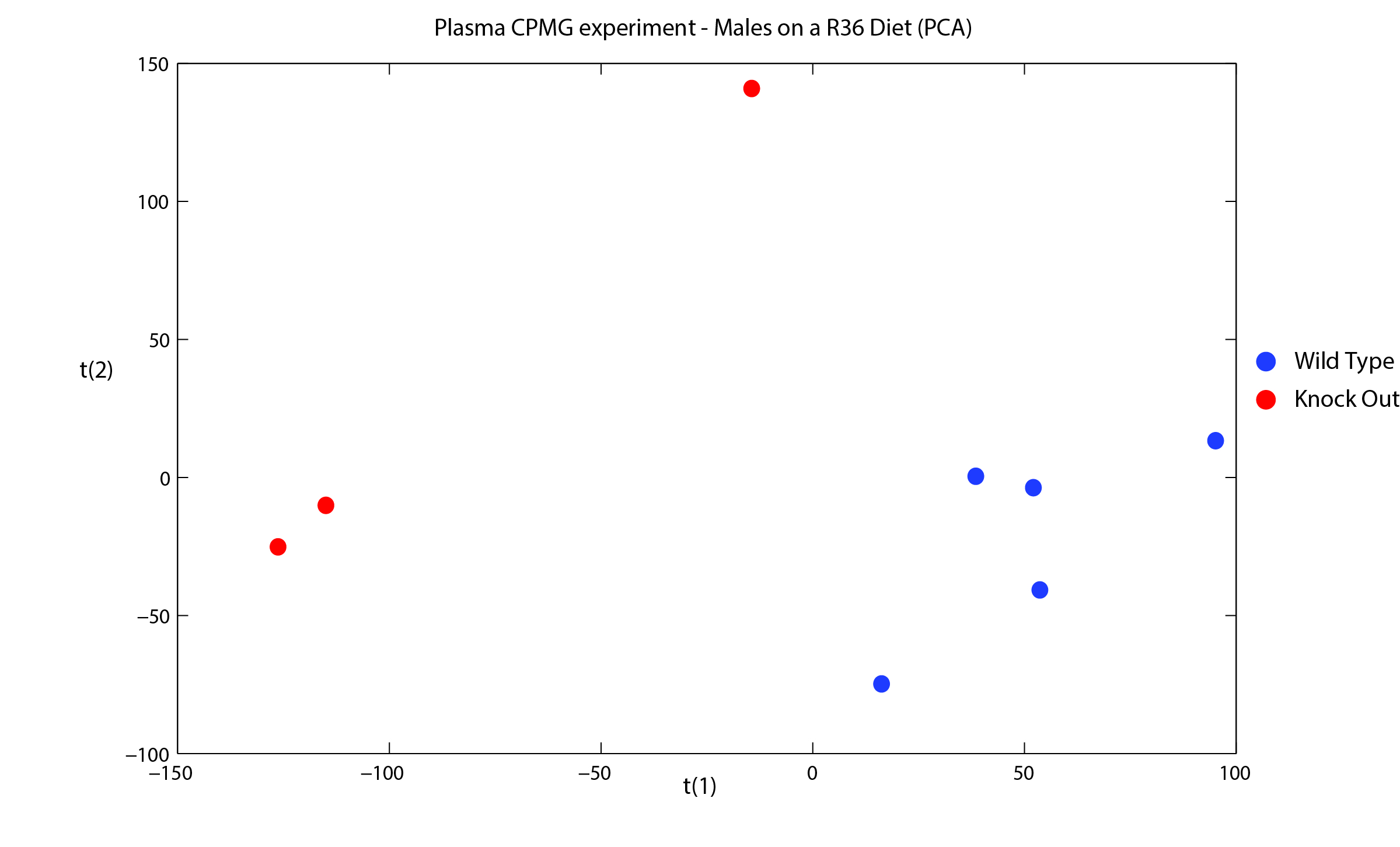
**a.**

**b.**

**
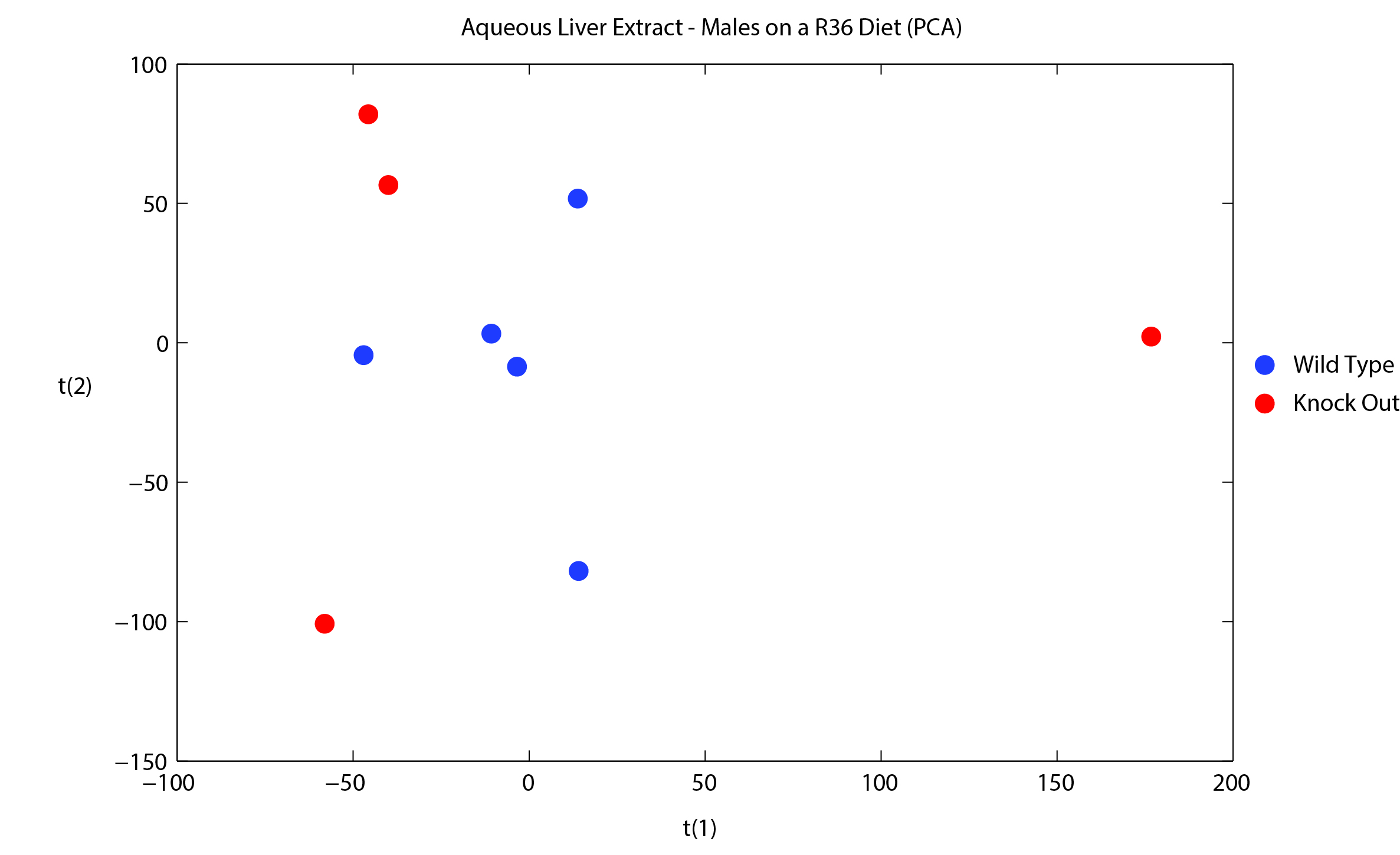
**


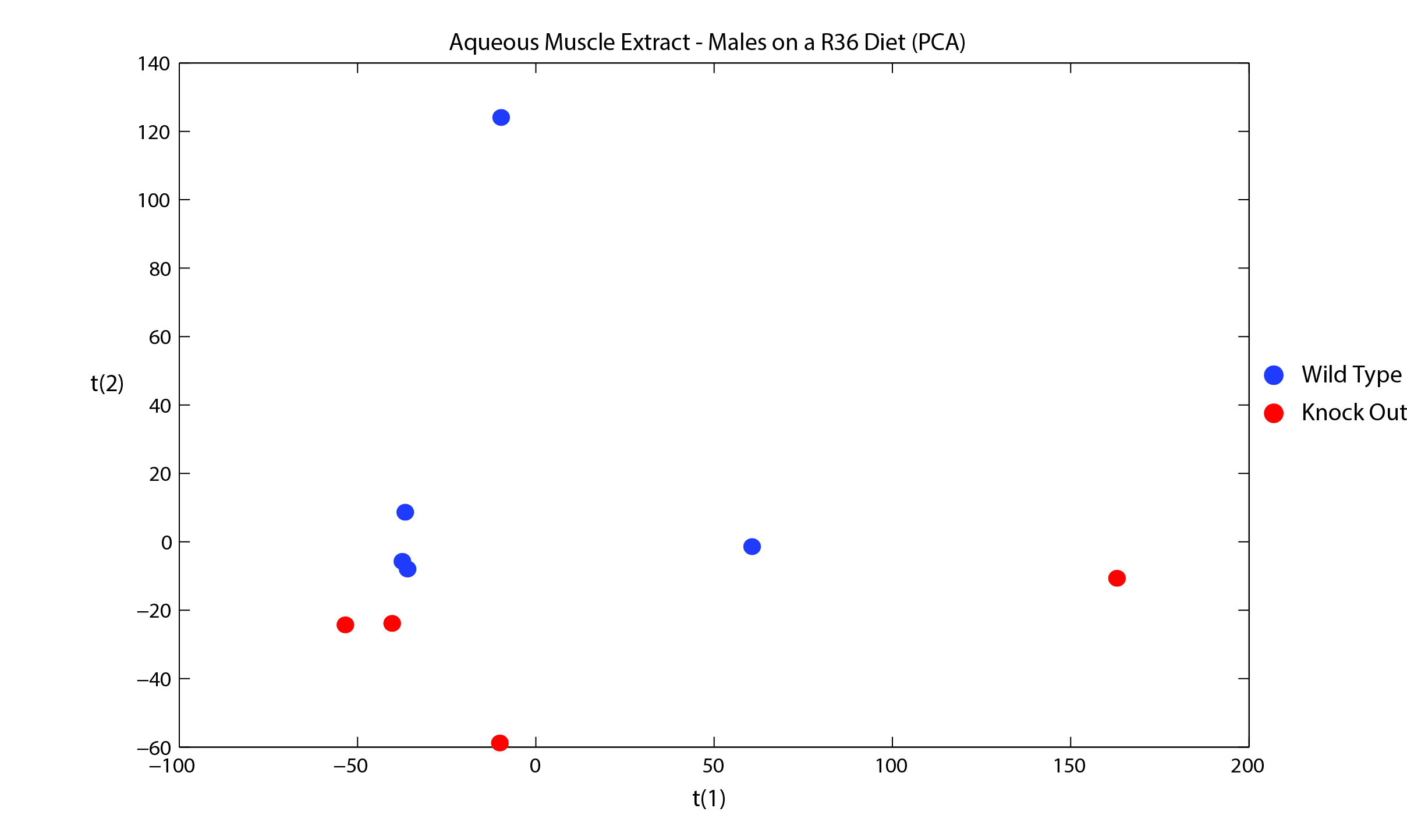
**c.**

**
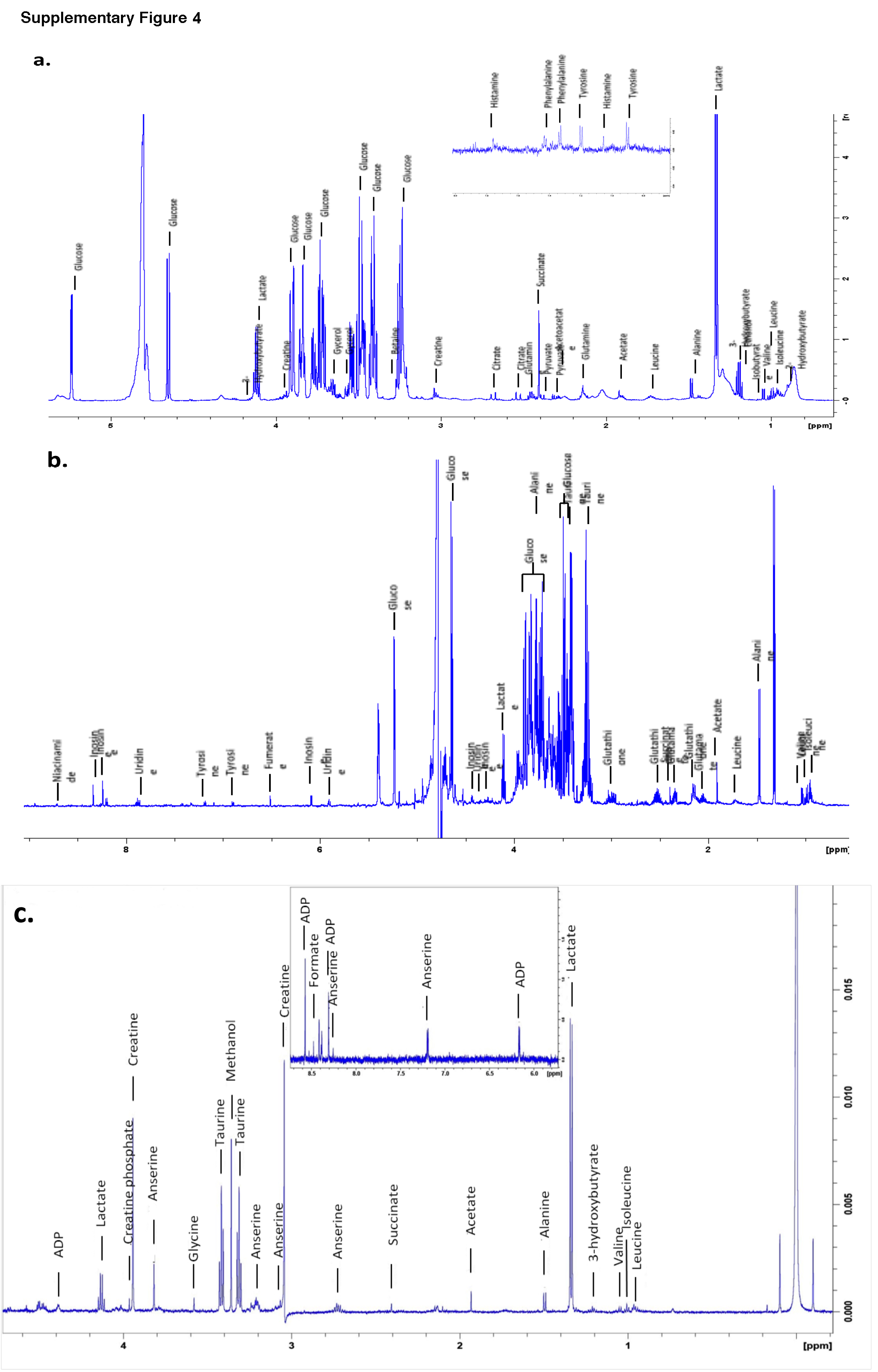
**

**
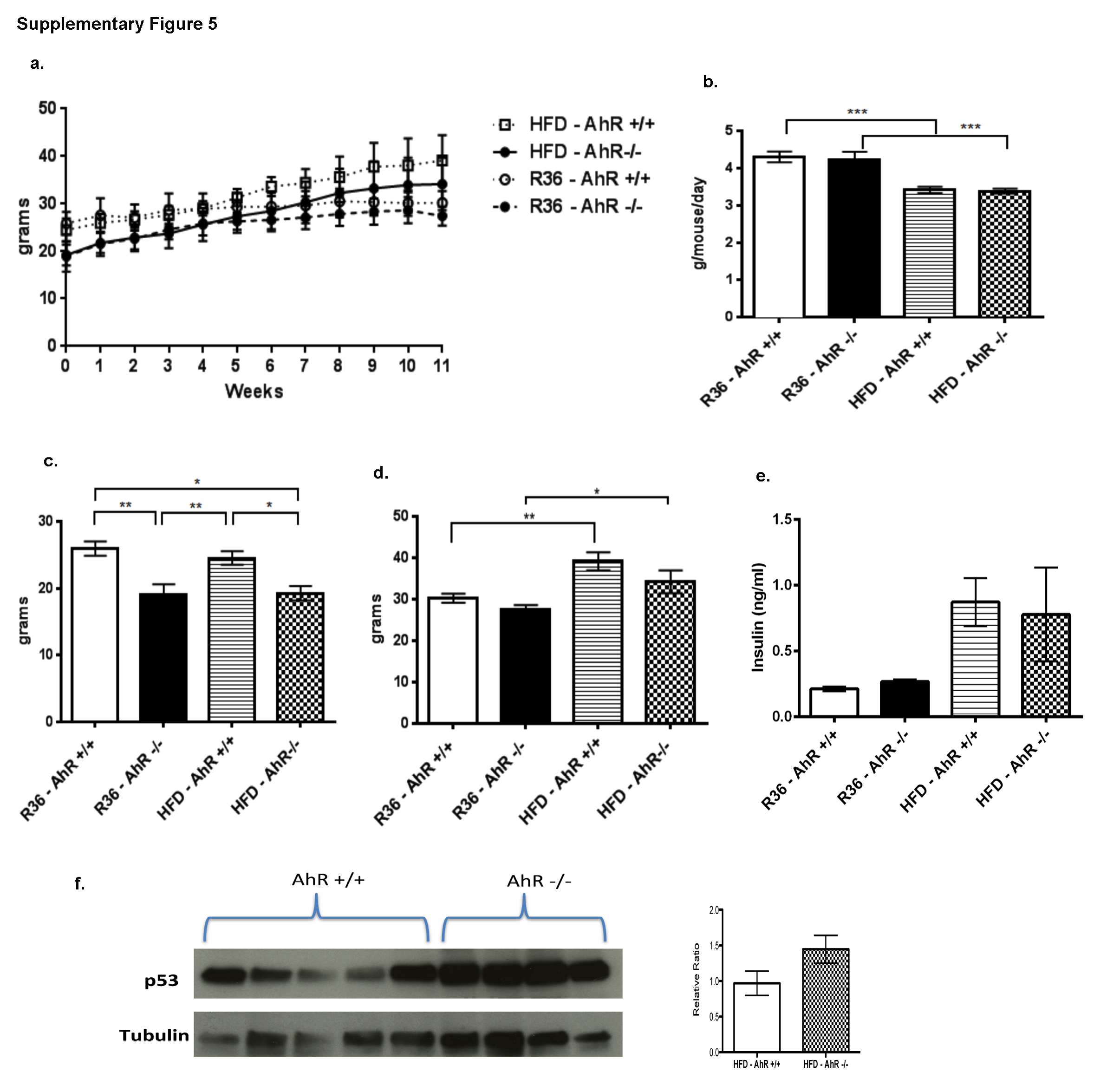
**

**Supplementary Table 1.** Average prevalence of distinct bacterial groups in the small intestine of AhR ^+/+^ and AhR ^-/-^ mice obtained by 454 sequencing platform. Mice received semi-synthetic diet without any AhR ligands (F2) or enriched in a known dietary AhR ligand (DIM) (n=4 AhR^-/-^ DIM-fed mice, n=4 AhR^-/-^ F2-fed mice, n=5 AhR^+/+^ DIM-fed mice, and n=6 AhR^+/+^ F2-fed mice).

|  | **F2** | | **DIM** | |
| --- | --- | --- | --- | --- |
| **Phylum** | **AhR +/+** | **AhR -/-** | **AhR +/+** | **AhR -/-** |
| **Bacteria; Other** | 0,35 | 0,29 | 0,04 | 0,24 |
| **Actinobacteria** | 2,73 | 0,35 | 0,14 | 0,93 |
| **Bacteroidetes** | 27,77 | 3,69 | 1,86 | 6,15 |
| **Cyanobacteria** | 0,03 | 0,02 | 0,03 | 0,01 |
| **Firmicutes** | 67,41 | 95,38 | 97,71 | 91,90 |
| **Proteobacteria** | 0,02 | 0 | 0,06 | 0,04 |
| **Tenericutes** | 1,69 | 0,27 | 0,15 | 0,73 |
|  |  |  |  |  |

|  |  | **F2** | | **DIM** | |
| --- | --- | --- | --- | --- | --- |
| **Phylum** | **Class** | **AhR +/+** | **AhR -/-** | **AhR +/+** | **AhR -/-** |
| **Other** | Other | 0,29 | 0,29 | 0,04 | 0,24 |
| **Actinobacteria** | Actinobacteria | 2,43 | 0,41 | 0,14 | 0,93 |
| **Bacteroidetes** | Bacteroidia | 24,04 | 3,80 | 1,86 | 6,15 |
| **Cyanobacteria** | Chloroplast | 0,12 | 0,02 | 0,03 | 0,01 |
| **Firmicutes** | Other | 0,10 | 0,34 | 0,01 | 0,12 |
|  | Bacilli | 56,87 | 88,12 | 96,26 | 82,96 |
|  | Clostridia | 14,60 | 6,69 | 1,44 | 8,82 |
| **Proteobacteria** | Alphaproteobacteria | 0 | 0 | 0,01 | 0 |
|  | Betaproteobacteria | 0,01 | 0 | 0 | 0,04 |
|  | Gammaproteobacteria | 0,01 | 0 | 0,05 | 0 |
| **Tenericutes** | Other | 0,01 | 0 | 0,01 | 0 |
|  | Erysipelotrichi | 1,30 | 0,28 | 0,15 | 0,65 |
|  | Mollicutes | 0,21 | 0,02 | 0 | 0,09 |

**Supplementary Table 2**. Average prevalence of distinct bacterial groups in the faeces of AhR ^+/+^ and AhR ^-/-^ mice obtained by 454 sequencing platform. Mice received semi-synthetic diet without any AhR ligands (F2) or enriched in a known dietary AhR ligand (DIM) (n=4 AhR^-/-^ DIM-fed mice, n=4 AhR^-/-^ F2-fed mice, n=5 AhR^+/+^ DIM-fed mice, and n=6 AhR^+/+^ F2-fed mice).

|  | **F2** | | **DIM** | |
| --- | --- | --- | --- | --- |
| **Phylum** | **AhR +/+** | **AhR -/-** | **AhR +/+** | **AhR -/-** |
| Other | 1,17 | 0,90 | 1,80 | 1,24 |
| Actinobacteria | 0,94 | 0,49 | 1,54 | 1,12 |
| Bacteroidetes | 31,03 | 25,37 | 26,29 | 25,55 |
| Firmicutes | 64,02 | 71,56 | 65,23 | 70,82 |
| Proteobacteria | 0,004 | 0 | 0 | 0,018 |
| Tenericutes | 2,83 | 1,67 | 5,09 | 1,26 |
| Verrucomicrobia | 0,004 | 0,005 | 0,04 | 0 |

|  |  | **F2** | | **DIM** | |
| --- | --- | --- | --- | --- | --- |
| **Phylum** | **Class** | **AhR +/+** | **AhR -/-** | **AhR +/+** | **AhR -/-** |
| **Other** | Other | 1,12 | 0,90 | 1,80 | 1,24 |
| **Actinobacteria** | Actinobacteria | 0,94 | 0,49 | 1,54 | 1,12 |
| **Bacteroidetes** | Other | 0 | 0,005 | 0 | 0 |
|  | Bacteroidia | 31,03 | 25,37 | 26,29 | 25,55 |
| **Firmicutes** | Other | 0,37 | 0,35 | 0,58 | 0,43 |
|  | Bacilli | 52,57 | 58,44 | 37,51 | 54,06 |
|  | Clostridia | 11,07 | 12,77 | 27,13 | 16,33 |
|  | Alphaproteobacteria | 0 | 0 | 0 | 0,01 |
| **Proteobacteria** | Gammaproteobacteria | 0,004 | 0 | 0 | 0,006 |
| **Tenericutes** | Other | 0,03 | 0,03 | 0,04 | 0,018 |
|  | Erysipelotrichi | 1,18 | 0,44 | 3,65 | 0,79 |
|  | Mollicutes | 1,62 | 1,21 | 0,44 | 1,40 |
| **Verrucomicrobia** | Verrucomicrobiae | 0,004 | 0,005 | 0 | 0,04 |

**Supplementary Table 3.** Average prevalence of distinct bacterial groups in the colon of AhR ^+/+^ and AhR ^-/-^ mice obtained by 454 sequencing platform. Mice received semi-synthetic diet without any AhR ligands (F2) or enriched in a known dietary AhR ligand (DIM) (n=4 AhR^-/-^ DIM-fed mice, n=4 AhR^-/-^ F2-fed mice, n=5 AhR^+/+^ DIM-fed mice, and n=6 AhR^+/+^ F2-fed mice)..

|  | **F2** | | **DIM** | |
| --- | --- | --- | --- | --- |
| **Phylum** | **AhR +/+** | **AhR -/-** | **AhR +/+** | **AhR -/-** |
| Other | 1,45 | 1,43 | 1,19 | 1,61 |
| Actinobacteria | 0,73 | 0,37 | 1,40 | 0,51 |
| Bacteroidetes | 35,75 | 27,69 | 36,35 | 31,86 |
| Cyanobacteria | 0 | 0,006 | 0 | 0 |
| Firmicutes | 59,42 | 69,06 | 54,79 | 64,99 |
| Proteobacteria | 0 | 0,006 | 0 | 0 |
| Tenericutes | 2,65 | 1,43 | 6,25 | 1,02 |

|  |  | **F2** | | **DIM** | |
| --- | --- | --- | --- | --- | --- |
| **Phylum** | **Class** | **AhR +/+** | **AhR -/-** | **AhR +/+** | **AhR -/-** |
| **Other** | Other | 1,45 | 1,43 | 1,19 | 1,61 |
| **Actinobacteria** | Actinobacteria | 0,73 | 0,37 | 1,41 | 0,51 |
| **Bacteroidetes** | Bacteroidia | 35,75 | 27,69 | 36,35 | 31,86 |
| **Cyanobacteria** | Chloroplast | 0 | 0,006 | 0 | 0 |
| **Firmicutes** | Other | 0,34 | 0,43 | 0,24 | 0,74 |
|  | Bacilli | 23,23 | 39,91 | 43,33 | 38,79 |
|  | Clostridia | 35,84 | 28,71 | 11,23 | 25,45 |
| **Proteobacteria** | Epsilonproteobacteria | 0 | 0,006 | 0 | 0 |
| **Tenericutes** | Other | 0,03 | 0,01 | 0 | 0 |
|  | Erysipelotrichi | 1,56 | 0,81 | 5,42 | 0,58 |
|  | Mollicutes | 1,06 | 0,61 | 0,83 | 0,44 |

**Supplementary Table 4**. qPCR primer sequences

Mouse:

| gene | Forward | Reverse |
| --- | --- | --- |
| 18SrRNA | 5'-ATGCCAAATCTCTGCTGCAT-3' | 5'-TGCCTATCTTGCTGTTTTTGAA-3' |
| Acox | 5'-TAACTTCCTCACTCGAAGCCA-3' | 5'-AGTTCCATGACCCATCTCTGTC-3' |
| AhR | 5'-AGGACCAAACACAAGCTAGA-3' | 5'-TGGAGATCTCGTACAACACA-3' |
| AhRR | 5'-GCCAATGCTGTGTAATGAAG-3' | 5'-AACAGAGCACCAAGAAAACA-3' |
| B-actin | 5'-CTGTATTCCCCTCCATCGTC-3' | 5'-CCTCGTCACCCACATAGGAG'3' |
| CD36 | 5'-TTGTGGGGTTACAAAGATCCA-3' | 5'-AGGATAAAACACACCAACTGTGG-3' |
| Cpt1a | 5'-ATGCCAAATCTCTGCTGCAT-3' | 5'-TGCCTATCTTGCTGTTTTTGAA-3' |
| Cyp4a1 | 5'-CTCATTCCTGCCCTTCTCAG-3' | 5'-TCCCATTTTTGGACTTCAGC-3' |
| Cpt2 | 5'-CAGCACAGCATCGTACCCA-3' | 5'-TCCCAATGCCGTTCTCAAAAT-3' |
| Cyp1a1 | 5'-CAGGATGTGTCTGGTTACTTTGAC-3' | 5'-CTGGGCTACACAAGACTCTGTCTC-3' |
| Cyp1a2 | 5'-CCATGTGCTTTGGGAAGAACTT-3' | 5'-GTCCTTGCTGTTATTCACGATGTT-3' |
| Ido1 | 5'-CAAAGCAATCCCCACTGTATCC-3' | 5'-ACAAAGTCACGCATCCTCTTAAA-3' |
| Fabp1 | 5'-ATGAACTTCTCCGGCAAGTACC-3' | 5'-CTGACACCCCCTTGATGATGTCC-3' |
| Cyp1b1 | 5'-TTTCCTCCTATCTCCCCC-3' | 5'-TCAAATCACCGTCCCAGC-3' |
| G6Pase | 5'-ACTGTGGGCCATCAATCTCCTC-3' | 5'-CGGGACAGACAGACGTTCAGC-3' |
| Hmgcs | 5'-TATGGGCTTCTGTTCAGTCCA-3' | 5'-AGCACTGTTTTGACAGCCTTG-3' |
| HPRT | 5'-TCCTCCTCAGACCGCTTTT-3' | 5'-CCTGGTTCATCATCGCTAATC-3' |
| LC3b | 5’-CGTCCTGGACAAGACCAAGT-3' | 5’-ATTGCTGTCCCGAATGTCTC-3' |
| MCAD | 5'-GCTCGTGAGCACATTGAAAA-3' | 5'-CATTGTCCAAAAGCCAAACC-3' |
| P53 | 5’-CCCCTGTCATCTTTTGTCCCT-3’ | 5’-AGCTGGCAGAATAGCTTATTGAG-3’ |
| PGC1a | 5'-AAGGGTTCCCCATTTGAGA-3' | 5'-AGGGTTATCTTGGTTGGCTTT-3' |
| PPARa | 5'-ATGCCAGTACTGCCGTTTTC-3' | 5'-TCTTGGCATTCTTCCAAAGC-3' |

Human:

| gene | Forward | Reverse |
| --- | --- | --- |
| Cyp1a1 | 5'-CACCATCCCCCACAGACA-3' | 5'-ACAAAGACACAACGCCCCTT-3' |
| AhR | 5'-GGGCATTAAATCCTTCTCAG-3' | 5-CAGTGGCTTCTTCAATTCCT-3' |
| B-actin | 5'-CCTGGCACCCAGCACAAT-3' | 5'-GCCGATCCACACGGAGTACT-3' |
